# Social defeat induces depressive-like states and microglial activation without involvement of peripheral macrophages

**DOI:** 10.1186/s12974-016-0672-x

**Published:** 2016-08-31

**Authors:** Michael L. Lehmann, Hannah A. Cooper, Dragan Maric, Miles Herkenham

**Affiliations:** 1 Section on Functional Neuroanatomy, Intramural Research Program, National Institute of Mental Health, NIH, Bldg. 35, Rm. 1C911, Bethesda, MD 20892-3724 USA; 2 NINDS Flow Cytometry Core Facility, NIH, Bethesda, MD 20892 USA

**Keywords:** Stress, Microglia, Depression, Neuroimmune, Macrophage, Microglial proliferation

## Abstract

**Background:**

We are interested in the causal interactions between psychological stress and activity within different compartments of the immune system. Psychosocial stress has been reported to not only alter microglia morphology but also produce anxiety-like and depressive-like effects by triggering CNS infiltration of macrophages from the periphery. We sought to test these phenomena in a somewhat different but standardized model of chronic social defeat (SD) stress.

**Methods:**

We used a paradigm of dyadic home pairing of dominant and subordinate mice that has been validated to induce powerful anxiety-like and depressive-like effects manifested by behavior assessed in social tasks. We administered the SD stress for 3 days (acute SD) or 14 days (chronic SD) and looked for monocyte entry into the brain by three independent means, including CD45 activation states assessed by flow cytometry and tracking fluorescently tagged peripheral cells from *Ccr2*^*wt/rfp*^ and *Ubc*^*gfp/gfp*^ reporter mice. We further characterized the effects of SD stress on microglia using quantitative morphometric analysis, ex vivo phagocytosis assays, flow cytometry, and immunochemistry.

**Results:**

We saw no evidence of stress-induced macrophage entry after acute or chronic defeat stress. In comparison, brain infiltration of peripheral cells did occur after endotoxin administration. Furthermore, mutant mice lacking infiltrating macrophages due to CCR2 knockout developed the same degree of chronic SD-induced depressive behavior as wildtype mice. We therefore focused more closely on the intrinsic immune cells, the microglia. Using *Cx3cr1*^*wt/gpf*^ microglial reporter mice, we saw by quantitative methods that microglial morphology was not altered by stress at either time point. However, chronic SD mice had elevated numbers of CD68^hi^ microglia examined by flow cytometry. CD68 is a marker for phagocytic activity. Indeed, these cells ex vivo showed elevated phagocytosis, confirming the increased activation status of chronic SD microglia. Finally, acute SD but not chronic SD increased microglial proliferation, which occurred selectively in telencephalic stress-related brain areas.

**Conclusions:**

In the SD paradigm, changes in CNS-resident microglia numbers and activation states might represent the main immunological component of the psychosocial stress-induced depressive state.

## Background

Converging clinical and experimental data support a role for the immune system in the etiology and maintenance of depressive disorders. Psychosocial stressors, which contribute to the development of affective disorders in humans, induce central and peripheral immune pathway signaling that is increasingly thought to be relevant to the pathophysiology of depression [1–4]. Stressors and associated neural activity in limbic brain areas, via activation of the sympathetic nervous system (SNS) and hypothalamic-pituitary-adrenal (HPA) axis, can alter the activation states of peripheral immune cells and trigger the release of proinflammatory cytokines that are thought to be pro-depressive through an incompletely understood central action [4–6]. The blood-brain barrier (BBB) effectively blocks the entry of both immune cells and cytokines into the brain, and thus there are many unanswered questions about how peripheral immune signals gain access to the brain and influence brain areas processing affective information. Several pathways for humoral signaling across the BBB have been identified [7], but cellular crossing may not occur unless the BBB is compromised [8]. Surprisingly, however, the entry of macrophages into the brain has recently been reported in several stress models [9–11].

Alternatively, a stress-induced inflammatory response in the brain can be more direct. Stress can precipitate the production of cytokines in the brain through activation of microglia, the resident CNS immune cells [12, 13]. Microglia are dynamic players serving to maintain proper neuronal function by sculpting synapses and clearing neuronal debris [14, 15]. At rest, they are highly motile, and their processes scan for signals that threaten local homeostasis [12, 16–18]. Upon the appearance of “activating” signals (such as purines, proinflammatory cytokines, glutamate receptor agonists, cell necrosis factors, trauma, or “danger” signals) [19] or the loss of constitutive “calming” signals, microglia rapidly alter their structure, morphology, and function [20–22]. Activated microglia can produce proinflammatory cytokines; present antigen, signaled by upregulation of major histocompatibility complex class II; and become phagocytic, signaled by upregulation of macrosialin (cluster of differentiation 68 (CD68)) [23, 24]. Excessive and/or prolonged microglial activation has been associated with the development of a number of pathologies [25–27], notably those associated with depressive states [25, 28, 29]. Most of our understanding of microglial activation comes from studies of pathological insults to the brain [26]. Classical accounts of microglial activation describe thickening and retraction of processes as the cell assumes a rounded, amoeboid shape. This might occur after diffuse activation and during phagocytosis, whereas directional process extension might occur in response to focal activation [30]. Whether these kinds of changes occur following non-pathogenic stressors is debated. Finally, microglia can proliferate, apparently in response to local demands [31, 32] and have been reported to do so after chronic unpredictable stress [33].

Our group is investigating several aspects of immune-brain interactions using a social defeat (SD) model of psychosocial stress (e.g., [34]). The SD paradigm reliably produces anxiety and depressive-like behavior [34–38]. Here we used acute and chronic SD to characterize several features of stress-induced immune responses. To gain accurate information about microglial numbers and morphology, we used the *Cx3cr1*^*wt/gfp*^ mouse, whose microglia strongly display green fluorescent protein (GFP) [16]. We also examined microglial activation status by counting CD68^hi^ cells by flow cytometry and measuring their phagocytic potential ex vivo. To assess macrophage entry into the brain, we used the *Ccr2*^*wt/rfp*^ mouse [39], whose activated macrophages are clearly distinguished by red fluorescent protein (RFP). We also tracked macrophages after adoptive transfer of GFP+ immune cells from a ubiquitous reporter mouse, *Ubc*^*gfp/gfp*^. Importantly, macrophage entry was recently reported in a similar model of psychosocial stress called repeated social defeat (RSD) [10].

The CNS stress response is driven by activity in the HPA axis, and the hypothalamic paraventricular nucleus (PVN) is the nodal point in the axis. It is controlled by the rodent medial prefrontal cortex (mPFC) [2]. Stress exerts structural changes on limbic brain areas such as the mPFC, amygdala, and hippocampus [40], the same areas that are associated with depression [41]. Some reports show that stress activates microglia in the PFC and hippocampus [10, 42]. Using an automated methodology, we quantified how SD alters several morphological properties of microglia in the mPFC, hippocampus, and PVN.

## Methods

### Animals

All procedures were approved by the National Institute of Mental Health Institutional Animal Care and Use Committee and conducted in accordance with the National Institutes of Health guidelines. Experiments were performed using CD-1 and C57BL/6J mice (NIH/NCI/Division of Cancer Treatment, Frederick, MD) and *Cx3cr1*^*wt/gpf*^ mice (Jackson model B6.129P-*Cx3cr1*^*tm1Litt*^/J) backcrossed onto a C57BL/6J background, *Ccr2*^*wt/rfp*^ mice (Jackson model B6.129(Cg)-*Ccr2*^*tm2.1Ifc*^/J), *Ubc*^*gfp/gfp*^ mice (Jackson model C57BL/6-Tg(UBC-GFP)30Scha/J), and interbred *Ccr2*^*wt/rfp*^*Cx3cr1*^*wt/gpf*^ mice. CD-1 mice used as aggressors in this experiment were retired 4–6-month-old breeders.

All animals were male and were housed in a pathogen-free environment on a 12-h light/dark cycle with lights off at 9:00 AM. Food (NIH-07 formulation; LabDiet, USA) and water were provided ad libitum. All cages were provided with hardwood bedding material (BetaChip; NEPCO). All environmental conditions were kept constant during the experiment. Testing was done in the dark phase in mice aged 8–10 weeks.

### Social defeat

Aggressor CD-1 male mice were single-housed in a large polycarbonate cage (24.0 × 46.0 × 15.5 cm; Lab Products) for 2 weeks with bedding incompletely refreshed once per week. The experimental intruder C57BL/6J wildtype or mutant mouse was subsequently placed into the resident CD-1 mouse’s home cage into which a 5.5-mm-thick perforated transparent polycarbonate partition had been placed down the middle to separate the pair. Circular 4.8-mm-diameter perforations, spaced equidistantly 7.9 mm apart in a grid pattern, encompassed the entire area of the partition. At this point, mandibular incisors of the CD-1 mouse were trimmed with blunt scissors to prevent wounding injuries to the subordinate mouse during the experiment. Defeats commenced after a 2-day accommodation period. The partition was removed for 5 min/day at approximately 11:00 A.M. to allow agonistic encounters between the mice. Defeat sessions were monitored to ensure that defeats, as previously described [38], reliably occurred. Although the partition physically separated the mice between defeat sessions, it allowed olfactory, visual, and auditory communication between the pair. The 24 h/day dyadic social housing exposed the defeated mouse to continuous psychological stress via sensory interaction with the aggressor. Experimental mice were exposed to social defeat (SD) for either 3 days (acute SD) or 14 days (chronic SD) from the same CD-1 mouse during the entirety of the experiment. Mice were killed 2 h after the last defeat for all experiments and tissues harvested. Homecage (HC) control mice were pair-housed in a divided 14.0 × 35.5 × 13.0 cm polycarbonate cage (Tecniplast) with one mouse on each side of the perforated divider.

### Behavioral analysis

One day prior to harvest, SD and HC mice were tested for affective changes using two tests that assess antisocial, anhedonic, and depressive-like behaviors. Four hours separated each test. Automated tracking and scoring of behavior (TopScan; Cleversys) was done as previously described [35, 43].

#### Social interaction

Mice were placed in a 50 × 50 cm open field arena containing two perforated Plexiglas cylinders (10 cm diameter). Plexiglas cylinders were placed in opposing corners; the walls of the cylinder were 10 cm from two adjacent edges of the arena wall. One cylinder contained an unfamiliar aggressor CD-1 mouse, and the other was empty. Test mice were placed in the open field and allowed to explore for 30 min. Social interaction (SI) quotients were defined as a ratio of duration investigating the cylinder with versus without the CD-1 mouse.

#### Urine scent marking

Briefly, and described in greater detail previously [36], 0.1 ml of estrous female mouse urine was blotted onto a 45.7 cm × 45.7 cm acid-free paper sheet (Strathmore Sketch Paper 400 series, SLS Arts) that was previously placed into an open field box (46 cm × 46 cm). A 2-cm-diameter spot of female urine was deposited in one corner of the arena, 10 cm from two adjacent edges of the paper. After the female urine was absorbed by the paper and dried (~1 min), male mice were placed in the center of the arena and allowed to freely explore and scent mark for 10 min. Sheets were air-dried at least 1 h and then sprayed with ninhydrin (Tritech Forensics), which stains amines in urine purple, and dried overnight. Dried sheets were digitally photographed and analyzed using ImageJ software (http://rsb.info.nih.gov/ij/). Images were converted to binary. Circles 20 cm in diameter were digitally placed in each quadrant such that the edges of the large circle touched the two outer edges of the quadrant. This allowed one circle to be centered on the female urine spot. The area of male urine marking within each of the four circles was measured, as was the total area of marks within the arena. Preference of urine marking was calculated by dividing the area of urine marks in a 20-cm circle by the total area of urine marks within the arena and then multiplied by 100.

### Immunohistochemistry

SD and HC mice were anesthetized with isoflurane and perfused transcardially with 0.9 % saline followed by ice-cold phosphate-buffered 4 % paraformaldehyde. Brains were removed and postfixed in the same fixative overnight followed by 25 % sucrose in phosphate-buffered saline (PBS) for 24 h. Coronal brain slices (30 μm thick) were collected on a freezing microtome. For cell counting experiments and for morphometric analysis, sections were counterstained with DAPI and mounted and dried on gelatin-coated slides. For studies examining cell proliferation, free-floating sections were mounted and dried on Superfrost slides (IHC World), subjected to epitope retrieval (IHC World) (15 min at 95 °C), washed, blocked in 4 % normal goat serum, incubated overnight at 4 °C in monoclonal rat anti-GFP antibody (ab290, Abcam) and mouse anti-proliferating cell nuclear antigen (PCNA) (sc-56, Santa Cruz). Slides were rinsed and incubated for 60 min in the appropriate secondary antisera (Alexa-488 for GFP and Alexa-555 for PCNA; Life Technologies). Hoechst 33342 was used to counterstain. In studies examining the presence of peripheral monocytes, free-floating sections were blocked, incubated overnight with anti-RFP (ab62341, Abcam) and anti-GFP (ab13970, Abcam), washed then incubated in secondary antisera directed against the host species (Alexa-488 for GFP and Alexa-555 for RFP) then counterstained with Hoechst 33342. Sections were analyzed on a Zeiss 780 confocal microscope.

### Automated morphometric characterization of microglia

Several reports have provided intriguing evidence that microglia alter their morphology in response to psychological stress and that these structural changes are considered a proxy for cell activation status. While informative, these studies relied either on subjective scoring or on manual threshold processing of immunostained tissue, a technique susceptible to bias and staining variables. To circumvent these issues, we examined computer-defined features of microglial morphology in *Cx3cr1*^*wt/gfp*^ mice using an automated image analysis method developed by Kozlowski and Weimer [44]. All morphometric segmentation was based on fluorescent signals from endogenous GFP in the microglia, removing any immunostaining variables. *Cx3cr1*^*wt/gfp*^ mice exposed to HC, acute SD, or chronic SD were used for this study. An additional group of HC mice was treated i.p. with 2 mg/kg of lipopolysaccharides from *Escherichia coli* 05:B55 (LPS; L2880, Sigma) in 0.9 % saline, a dose previously shown to activate microglia in vivo [44], and processed as above after 48 h for histological analysis.

Coronal sections containing regions of interest were identified. These regions included the infralimbic and prelimbic regions of the medial prefrontal cortex (PFC) in the three or four sections closest to +1.70 mm from the bregma, the hypothalamic paraventricular nucleus (PVN) in sections between −0.70 and −0.94 mm from the bregma, and the dorsal hippocampus (−0.9 to −2.4 mm from the bregma). Areas of interest were scanned on a Zeiss 780 confocal microscope, and 10 optical sections, 1 μm apart (0.42 μm/pixel resolution), were captured from the middle of 30-μm-thick tissue. 2D maximum intensity projections (MIPs) of 3D image data were then generated. Automatic cell segmentation from 2D MIPs was performed using MATLAB (code kindly provided by Cleopatra Kozlowski, Genentech). Briefly, and described in detail previously [44], microglia cell positions were identified in each MIP using a regional maxima finding algorithm. An iterative threshold segmentation routine was then applied around the region of each cell position to generate a cell mask. Post-segmentation testing was then used to verify that each cell mask accurately represented a single cell with a single soma. Once segmented, each cell mask provided the spatial coordinate of individual microglia. The shape of each mask was then described using several morphometric measures. These include cell perimeter length (the length around the periphery of each cell), cell spread (the average distance from the cell center of mass to its detected extremes), eccentricity (the ratio of the major axis to the minor axis of the smallest circle that can fit the extensions of the cell), roundness (4π × area/cell perimeter length^2^), and soma size (the area contained within the soma mask). All parameters were calculated from individual segmented soma or cell masks using the MATLAB regionprops function with the properties specified as perimeter, extrema, eccentricity, and area, respectively. For statistical purposes, these six morphometric parameters are treated as independent measures and are not compared against each other. Reliability of the MATLAB code was tested on 20 randomly picked images prior to running the code through the entire dataset of confocal imagery. Here, MATLAB code was validated against measurements obtained from manual counting. Manual counting was achieved using tools present in NIH ImageJ.

### Flow cytometry

Whole brains minus cerebellum were dissected from HC and SD mice perfused with 0.9 % saline. Single-cell suspensions were created through enzymatic digestion using the Neural Tissue Dissociation Kit (Miltenyi Biotec) for 35 min at 37 °C. Further processing was performed at 4 °C. Tissue debris was removed by passing the cell suspension through a 40-μm cell strainer. Myelin was removed using a discontinuous Percoll gradient. After enzymatic dissociation, cells were resuspended in 70 % Percoll layered under 30 % Percoll and banded on a 70 % Percoll cushion at 1000×*g* at 10 °C for 40 min. The supernatant containing myelin was removed, and cells were collected at the 30–70 % Percoll interface. Cells isolated from wildtype mice were labeled by incubating on ice for 30 min with anti-mouse CD16/CD32 (Clone 2.4G2, BD Pharmingen) to block Fc receptors, and then incubated on ice for 30 min with a mix of fluorochrome-conjugated anti-mouse antibodies: CD11b-APC (Miltenyi Biotec), CD45-PE (Biolegend), and CD68-PerCP/Cy5.5 (Biolegend). Brain cells isolated from interbred *Ccr2*^*wt/rfp*^*Cx3cr1*^*wt/gfp*^ mice were enumerated without further fluorescent enhancement (as is).

In adoptive transfer experiments, the spleens were also enumerated for GFP+ cells to determine successful colonization. The spleens were removed prior to perfusion, and single-cell suspensions were created using a gentleMACS dissociator (Miltenyi Biotec). Tissue debris was removed by passing the cell suspension through a 40-μm cell strainer, and cells were concentrated using a discontinuous Percoll gradient. Cells were resuspended in 70 % Percoll layered under 30 % Percoll and banded on a 70 % Percoll cushion at 1000×*g* at 10 °C for 40 min. Cells at the 30–70 % Percoll interphase were examined for GFP. Flow cytometry was done on a MoFlo Astrios (Beckman Coulter), and data were analyzed with FlowJo software (TreeStar).

### Microglia isolation

Microglia were isolated from the brains of HC and SD mice using enzymatic digestion and Percoll gradients in a manner similar to that used for flow cytometry. After banding on 30/70 Percoll gradients, cells were magnetically labeled with CD11b microbeads (Miltenyi Biotec). Using the manufacturer’s guidelines, CD11b^hi^ cells were separated in a magnetic field using MS columns (Miltenyi Biotec). Each brain extraction yielded approximately 3 × 10^5^ viable cells. Cells were used for phagocytosis assays.

### Ex vivo microglia phagocytosis assay

Microglia isolated with CD11b microbeads from the brains of HC and SD mice were suspended in DMEM F12 + GlutaMax supplemented with 10 % qualified fetal bovine serum and plated at 5 × 10^4^ cells/well on Lab-Tek Chamber slides (Nalge Nunc) for 24 h at 37 °C and 5 % CO_2_. To determine the phagocytic ability of microglial cells, we chose apoptotic neural cells as targets, because they would closely approximate natural targets in vivo. Neural cells were isolated from the brains of HC mice using enzymatic digestion and Percoll gradients in a manner similar to flow cytometry experiments except cells were resuspended in 20 % Percoll and layered over 70 % Percoll. Neural cells were collected at the 20/70 % Percoll interphase, washed, resuspended in DMEM F12 + GlutaMax, and exposed to 254 nm ultraviolet (UV) irradiation for 20 min. Apoptosis was verified with trypan blue. UV-exposed cells were then stained with 5 μl of 5(6)-TAMRA, succinimidyl ester (Invitrogen), and washed thoroughly with cold PBS before they were fed to microglia previously incubated for 24 h. After 3 h at 37 °C and 5 % CO_2_, media was removed and cells washed and fixed with 4 % PFA for 20 min. Cells were blocked in 10 % goat serum in 0.1 M PBS containing 0.3 % Triton X-100 and 0.5 % BSA, followed by incubation with anti-CD11b (AbD Serotec, 1:1000) for 1 h at room temperature. Cells were washed for 10 min three times at room temperature in 0.1 % Triton X-100 (Sigma) in PBS, followed by incubation with Alexa-fluor 488 goat anti-rat IgG antibodies (1:1000, Invitrogen) for 1 h at room temperature. Cells were washed again with 0.1 % Triton X-100 in 0.1 M PBS (10 min, three times), counterstained with DAPI for 1 min, washed with PBS, and coverslipped with Fluoromount (Sigma). From each condition, 25 images were randomly captured with confocal microscopy. A phagocytic index was calculated by dividing the total area of phagocytosed TAMRA-labeled apoptotic cells by the total area of microglial cell (CD11b+) using ImageJ software.

### Adoptive transfer of GFP+ cells

It is possible that peripheral macrophages entering the brain can become indistinguishable from resident microglia due to CNS-induced alterations in cell surface markers normally used to distinguish macrophages from microglia. To address this possibility, spleen cells from *Ubc*^*gfp/gfp*^ mice with ubiquitous expression of GFP were adoptively transferred into wildtype mice to determine if peripheral monocytes are recruited into the brain after stress. The spleens from HC *Ubc*^*gfp/gfp*^ donor mice were dissociated using a gentleMACS dissociator (Miltenyi Biotec). Tissue debris was removed by passing the cell suspension through a 40-μm cell strainer, and cells were concentrated using a discontinuous Percoll gradient. Cells were resuspended in 70 % Percoll layered under 30 % Percoll and banded on a 70 % Percoll cushion at 1000×*g* at 10 °C for 40 min. Cells at the 30–70 % Percoll interphase were resuspended in physiological PBS and injected retro-orbitally at a concentration of 25 million cells per host in a volume of 0.15 ml. All mice were sacrificed 3 days after transfer. As diagrammed in Fig. 5a, three transfer and treatment conditions were used to test peripheral monocyte infiltration: (1) mice received cell transfer after 11 days of SD then exposed to a further 3 days of SD (chronic SD), (2) HC mice were exposed to 3 days of SD after transfer (acute SD), or (3) HC mice received transfer 3 days prior to endpoint analysis (HC control). As a positive control for demonstrating splenocyte infiltration into the brain, mice received cell transfer, and then 0.1 mg/kg LPS was administered i.p. followed by 100 ng of IL-1β (R&D, cat. #401-ML) administered intracisternally into the CSF. The treatments occurred at 24-h intervals (Fig. 5a).

### Intracisternal IL-1β injection

Mice were anesthetized with isoflurane and fixed in a stereotactic frame. For injection of IL-1β into the cisterna magna, the skin over the posterior atlanto-occipital membrane was cut, and the muscular layers were moved aside. A pulled glass pipette attached to the tip of a 10-μl Hamilton syringe (33-g needle) was carefully inserted through the dura into the cisterna magna, and 100 ng of IL-1β in 5 μl of 0.1 % mouse serum albumin and PBS was injected at the rate of 1 μl/min. The muscular layers and skin were closed by suture, and the mouse was kept on a heating pad and returned to the standard cage after it recovered from anesthesia.

### Statistics

Data for all experiments were analyzed using parametric statistics with ANOVA or multi-factor ANOVA as appropriate using SPSS software. ANOVA analysis was followed by post hoc tests or planned comparisons as projected from the design of each experiment. Bivariate correlations were determined using Spearman’s correlation coefficients. Bonferroni corrections for multiple comparisons were used where appropriate. Data were presented as mean ± SEM.

## Results

### Behavior after acute SD and chronic SD

**Fig. 1:**
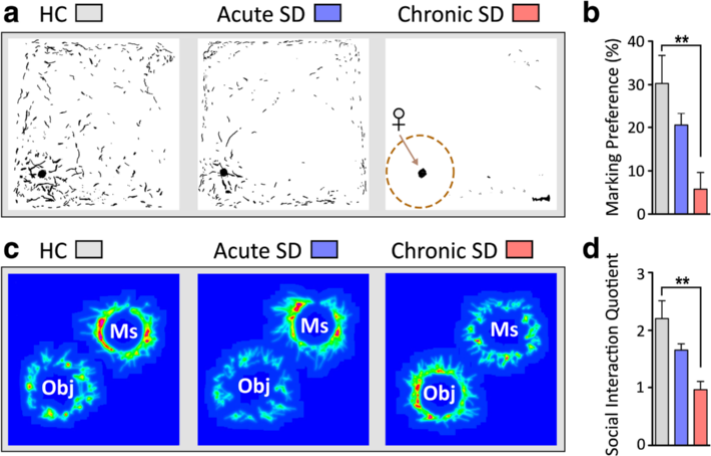
*Cx3cr1* ^*wt/gfp*^ mice exposed to chronic defeat show substantial declines in the expression of social and sexual behaviors compared to acute-stress and non-stressed homecage (HC) *Cx3cr1* ^*wt/gfp*^ mice. **a** Binary images of urine scent marks deposited by a male mouse housed either in HC or exposed to acute or chronic SD stress. Overlaid on the chronic SD image is a diagram showing the analyzed quadrant with female urine. Marking preferences are calculated by dividing the area of male urine marks within this quadrant by the total area of marks deposited in the arena. **b** Chronically stressed males show a sharp reduction in marking preference compared to homecage controls. **c** Heat maps of the social interaction test illustrating effects of chronic SD on social behavior. **d** Chronic SD mice spent significantly less time with the aggressor mouse in the social interaction test compared to non-stressed control mice. The social interaction (SI) quotient is determined by the proportion of time the experimental mouse explored an enclosure containing an aggressor mouse versus an empty enclosure. *Ms* mouse, *Obj* object. Results are expressed as mean ± SEM (*n* = 8 per group). One-way ANOVA followed by Bonferroni’s post hoc test **p < 0.01

The urine scent marking (USM) and social interaction (SI) tests were used to measure hedonic drive and sociability; declines in these behaviors are maladaptive responses, and they occur coincidently with anxiety-like and depressive-like behaviors measured in open field, light/dark box, elevated zero maze, sucrose preference test, forced swim test, and tail suspension test [35–38]. *Cx3cr1*^*wt/gfp*^ mice exposed to chronic SD showed significant reductions in marking preference (*F*_(2,23)_ = 7.26, *p* < 0.005) compared to HC and acute SD *Cx3cr1*^*wt/gfp*^ mice in the USM task (Fig. 1a, b). Similar behavioral trends were observed in SI tests; chronic defeat substantially reduced SI (*F*_(2,23)_ = 9.05, *p* < 0.001) compared to homecage and acutely stressed mice (Fig. 1c, d). Acute SD did not significantly alter behavior (*p* > 0.05) compared to HC mice. Thus, chronic SD but not acute SD had depressive-like effects in two tests of sociability.

### Absence of peripheral cell infiltration into brain after acute SD and chronic SD

**Fig. 2:**
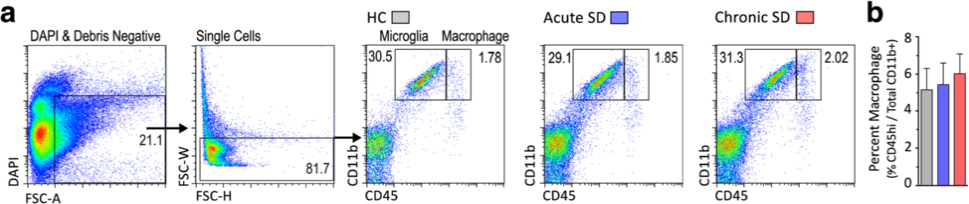
Social defeat does not increase macrophage cell trafficking to the brain. **a** Cell characterization strategy. Cells were first gated by DAPI exclusion and size to identify live cells, then gated to exclude doublets. Live single cells were then assessed for CD11b and CD45 staining. Representative bivariate dot plots show brain cells from HC, acute SD, or chronic SD mice stained and gated for microglia (CD11b^hi^ CD45^low^) and macrophages (CD11b^hi^ CD45^hi^). **b** Number of macrophages in the brain was similar between all treatment conditions. Results are expressed as percentage CD45^hi^ cells in the CD11b ^hi^ cell population (*n* = 8 per group). One-way ANOVA detected no significance between groups

As noted, repeated social defeat (RSD) increased the migration of myeloid-derived CD11b^hi^ cells from the periphery into the brain [10, 45]. As in those studies, we used flow cytometry to differentiate between resident microglia and CNS-infiltrating monocytes based on expression level of CD45 [46]. In wildtype C57BL/6J mice, brain microglia and macrophages were collected 2 h after the final social defeat. Figure 2a shows representative plots of CD11b^hi^ CD45^lo^ microglia and CD11b^hi^ CD45^hi^ macrophages. Neither acute SD nor chronic SD elevated numbers of CD45^hi^ macrophages in brain (Fig. 2b).

**Fig. 3:**
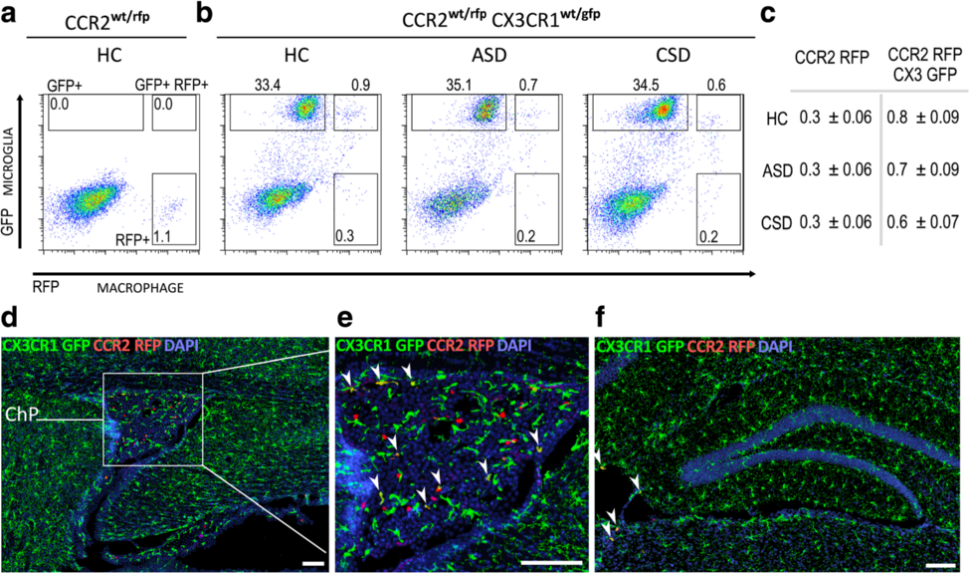
Stress does not cause extravasation of *Ccr2* ^*wt/rfp*^ macrophages (RFP, *red*) into the brain parenchyma. **a** Representative bivariate dot plot showing gating strategy for RFP in a HC control *Ccr2* ^*wt/rfp*^ mouse. **b** Bivariate dot plot showing CX3CR1-GFP+ microglia, CCR2-RFP+ macrophages, and CCR2-RFP CX3CR1-GFP dual positive cells in the brains of *CCR2* ^*wt/rfp*^ *CX3CR1* ^*wt/gfp*^ mice exposed to HC and acute (ASD) and chronic social defeat (CSD). **c** Average number of CCR2-RFP+ cells and CCR2-RFP+ CX3CR1-GFP+ double-positive cells detected in all conditions (mean ± SEM *n* = 6). **d** Peripheral CCR2-RFP+ macrophages are detected in the choroid plexus of *CCR2* ^*wt/rfp*^ *CX3CR1* ^*wt/gfp*^ mice, regardless of stress exposure. HC condition is shown. The *red* peripheral CCR2-RFP+ cells contrast with *green* CX3CR1-GFP+ microglia. **e** Magnified view of choroid plexus detailing RFP/GFP dual positive cells with *arrows*. **f** Peripheral CCR2-RFP+ macrophages (*red*) are detected in the leptomeninges but not in the brain parenchyma of the dorsal hippocampus. *ChP* choroid plexus. Six animals per group were examined. Scale bars: **d** *–* **f** = 100 μm

We wondered whether the CD45 expression level might be downregulated in monocytes following entry into the brain. Therefore, we used another method to track peripheral monocytes. The chemokine CCR2 characterizes an inflammatory subclass of bone marrow-derived monocytes with a high migratory and organ infiltration capacity [47] that is absent in all CNS-resident cell types [48]. We crossed *Ccr2*^*wt/rfp*^ mice with *Cx3cr1*^*wt/gfp*^ mice to monitor red fluorescent peripheral monocytes (CCR2-RFP+) and green fluorescent microglial cells (CX3CR1-GFP+) in the same animal. We exposed *Ccr2*^*wt/rfp*^*Cx3cr1*^*wt/gfp*^ mice to acute SD or chronic SD, and brains of these mice were analyzed by flow cytometry and immunochemistry for the presence of CCR2-RFP cells. Confirming our previous observations, no significant differences in numbers of double-positive CCR2-RFP+ CX3CR1-GFP+ cell populations or single-positive CCR2-RFP+ cell populations were detected under any condition (Fig. 3a–c). The data show that CCR2-RFP+ macrophages are present in very small numbers in the brain, but they do not migrate into brain in response to SD. These results were further confirmed through confocal microscopic analysis of limbic, hypothalamic, and cortical structures. CCR2-RFP+ and CCR2-RFP+ CX3CR1-GFP+ cells were detected in barrier locations such as the choroid plexus (Fig. 3d, e) and leptomeninges (Fig. 3f), and the number of cells in these regions was not significantly different between groups. Importantly, CCR2-RFP+ cells were absent from brain parenchyma in all examined regions (Fig. 3f).

**Fig. 4:**
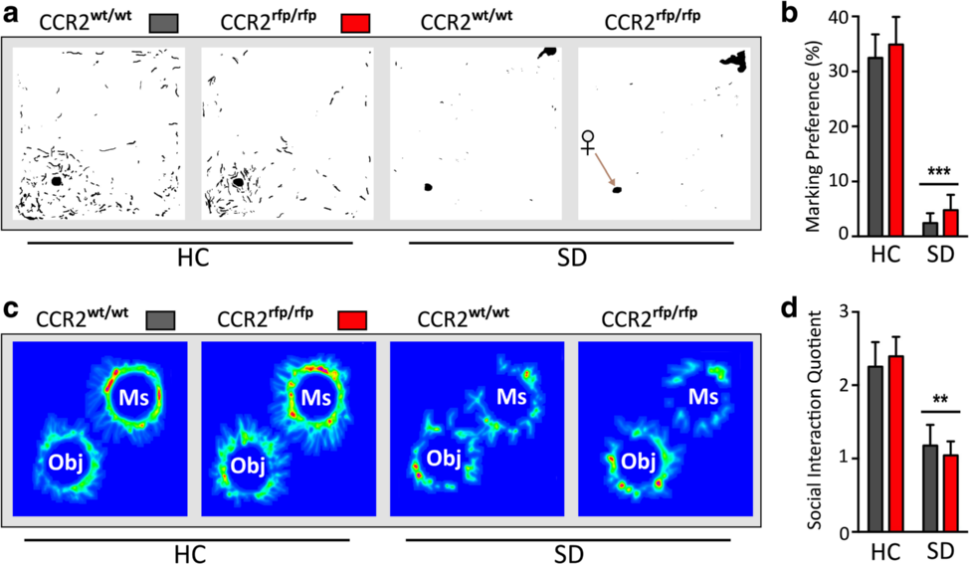
Chronic social defeat produces disrupted social scores in mice lacking CCR2+ monocytes. **a** Binary images of urine scent marks from *Ccr2* ^*wt/wt*^ mice and mice lacking CCR2-containing monocytes (*Ccr2* ^*rfp/rfp*^) exposed to homecage (HC) or chronic defeat stress (SD). **b** Defeated groups showed a sharp reduction in scent marking compared to HC groups; absence of CCR2-positive monocytes did not confer resilience to social defeat. **c** Heat maps illustrating the effects of social defeat on social behavior during the social interaction test. **d** Chronic SD mice spent significantly less time with the aggressor mouse in the social interaction test compared to non-stressed control mice; depressive-like behavior was comparable between genotypes. *Ms* mouse, *Obj* object. Results are expressed as mean ± SEM (*n* = 8 per group). One-way ANOVA followed by Bonferroni’s post hoc test **p < 0.01, ***p < 0.001

CCR2 is a chemokine receptor crucial for monocyte infiltration into inflamed tissue, and we tested whether knockout of CCR2 confers behavioral protection to chronically defeated mice. Homozygous *Ccr2*^*rfp/rfp*^ mice, which lack the monocyte population that extravasates into the brain, were placed into chronic SD. *Ccr2*^*rfp/rfp*^ and *Ccr2*^*wt/wt*^ mice showed similar and significant reductions in marking preference (stress effect: *F*_(1,28)_ = 19.61, *p* < 0.001) and social interaction (stress effect: *F*_(1,28)_ = 66.06, *p* < 0.001) after chronic SD compared to non-stressed cohorts. No effect of genotype was observed (*p* > 0.05) (Fig. 4). The result provides indirect support for the lack of CCR2^hi^ monocyte entry with chronic SD and underscores that these migratory cells do not contribute to altered affect in the chronic SD model.

**Fig. 5:**
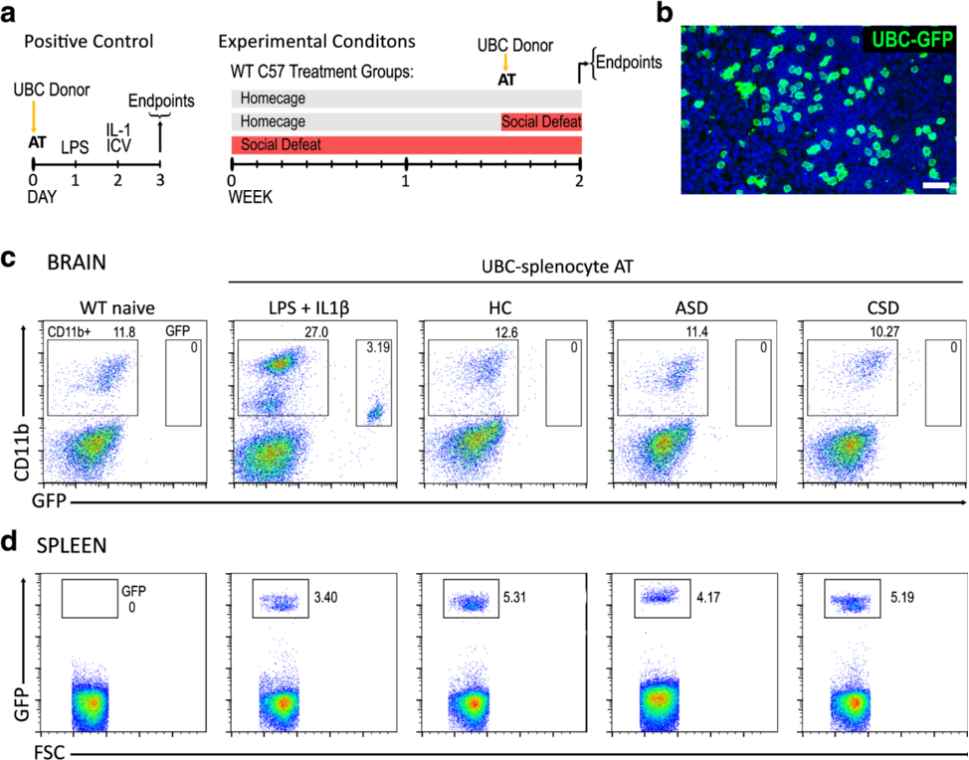
GFP+ splenocytes transferred from UBC-GFP mice do not extravasate into the brain of stressed wildtype mice. The experimental timeline is shown in (**a**) for positive control conditions and experimental conditions. Transferred GFP+ cells colonize the spleen of wildtype recipient (example is from a mouse in chronic SD) (**b**). Representative bivariate dot plots of brain cells isolated from wildtype naïve and from wildtype mice with adoptive transfer of UBC splenocytes (**c**). Cells are stained and gated for CD11b^hi^ cells and UBC-GFP+ cells. UBC-GFP+ cells are observed in the brains of mice that received inflammatory stimulus (LPS + 1L-1β) but not in experimental stress conditions. **d** Flow cytometry confirmed the presence of UBC-GFP+ cells in the spleens of wildtype mice receiving transfer of cells from *Ubc* ^*gfp/gfp*^ donors. (*n* = 4 per group). Scale bar = 20 μm

It is possible that peripheral monocytes become indistinguishable from resident microglia upon infiltration into the CNS. Furthermore, clear distinction of activated microglia from infiltrating monocytes is hampered by the overlap in the expression of macrophage-associated factors typically present also in microglia. To address these concerns, splenocytes from *Ubc*^*gfp/gfp*^ mice with ubiquitous cellular expression of GFP were adoptively transferred into wildtype mice to determine, definitively, if peripheral monocytes are recruited into brain after stress. A positive control group of HC mice was given a proinflammatory challenge that recruits peripheral immune cells into the brain. The mice first received UBC-GFP+ spleen cells, and 24 h later, they were administered LPS i.p. followed 24 h later by 1L-1β intracisternally (Fig. 5a). In this positive LPS + IL-1β control condition, there was a robust recruitment (approximately twofold increase) of CD11b+ cells, both unlabeled and GFP-labeled, into the brain, and ~10 % of CD11b+ cells were GFP+ (Fig. 5c). In contrast, no GFP+ cells were present in the brain in either the acute or chronic SD condition, and there were no increases in CD11b+ cell numbers (Fig. 5c). GFP+ cells were present in the spleens of all animals given adoptive transfer demonstrating healthy colonization of the transferred cells (Fig. 5b, d). Experimental stress conditions had no effect on the percentage of GFP+ cells present in the spleens of recipients (*p* > 0.05; *n* = 4 per group).

### Automated morphometry-based quantification of microglia activation

**Fig. 6:**
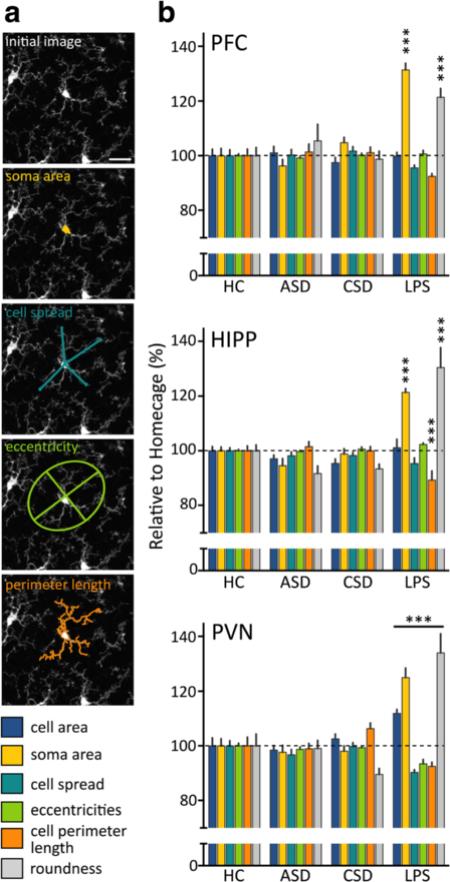
Microglia show regionally specific morphological changes to LPS, but not to SD stress in CX3CR1^WT/gfp^ mice. **a** Panel provides examples of four morphological parameters used to characterize the shape of segmented cells: soma size, cell processes spread, eccentricity, and cell perimeter length. Initial image is provided for comparison. Scale bar = 20 μm. **b** Graphs show relative changes in microglia morphometric parameters in the prefrontal cortex (PFC), hippocampus (HIPP), and paraventricular nucleus (PVN) of the hypothalamus of mice exposed to acute SD (ASD), chronic SD (CSD), or 2 mg/kg LPS. Significant changes occur only in mice exposed to LPS, and these effects are region-dependent. For each parameter, values represent mean fold change ± SEM compared to HC (*n* = 10 for HC, ASD, and CSD groups, and *n* = 6 for the LPS group). One-way ANOVA followed by Bonferroni’s post hoc test was used for each morphometric parameter in each area. Bonferroni correction for multiple comparisons has been applied. *** Indicate statistical significance compared to all other groups *p* < 0.001

We used a previously validated [44] automated segmentation and morphometric analysis method to quantitatively assess cell morphology (Fig. 6a). *Cx3cr1*^*wt/gfp*^ mice were exposed to HC, acute SD, or chronic SD. A subset of HC mice was injected with 2 mg/kg LPS prior to collection in order to assess the morphometric analysis capabilities of this method. For each brain region, the calculated morphometric parameters of each condition were normalized to HC condition values and plotted as a percentage, where the mean values in the control sample are defined as 100 %. Stress has little effect on any of the six morphometric parameters examined. In striking contrast, LPS exerted substantial effects on microglial morphology, and the magnitude and type of changes were dependent on the anatomical location (as detailed in the ANOVA table, Table 1). For instance in the PFC, LPS increased both roundness and soma size (131.3 and 121.4 %, respectively) compared to all other treatment conditions. In the hippocampus, LPS altered not only soma size and roundness, but it also significantly reduced cell perimeter length. The most robust effects were observed in the PVN (Fig. 6b). LPS significantly altered all six morphometric parameters; both the cell size and soma size were elevated as was roundness, but the spread of cell extremities, cell eccentricity, and perimeter were significantly reduced. Although soma area and roundness were the measures most sensitive to LPS, changes in cell processes spread and eccentricity suggest that this technique is sensitive to subtle changes in soma size and shape as well as process architecture. Importantly, the data highlight regional heterogeneity of microglial responses and suggest that differences in immune vigilance may be influenced by local microenvironments within specific brain regions.

**Table 1:** *F* values obtained from ANOVA of each microglial morphometric parameter in each brain area

Brain area	# of cells	Morphometric parameter		ANOVA *F*_(3,32)_, *p*			
		Cell area	Soma area	Cell spread	Eccentricity	Perimeter	Roundness
PFC	2909		19.65, *p* < 0.0001				2.75, *p* < 0.05
HIPP	2226		11.37, *p* < 0.0001			3.14, *p* < 0.05	18.22, *p* < 0.0001
PVN	1581	4.15, *p* < 0.01	14.68, *p* < 0.0001	3.97, *p* < 0.02	6.32, *p* < 0.001	4.17, *p* < 0.01	3.67, *p* < 0.05

One-way ANOVA examined the effects of acute or chronic social defeat exposure, or LPS on microglia morphology in three brain regions. Only statistically significant effects and interactions are included. Nonsignificant analyses have been left blank

### Microglial activation status

**Fig. 7:**
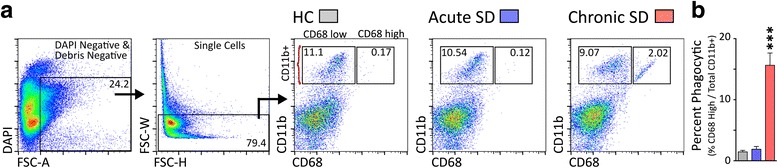
Chronic social defeat increases the number of CD68^hi^ cells. **a** CD68 staining was used as a proxy for phagocytic activity. Gating strategy used to examine single live cells for the co-expression of CD11b and CD68. Representative bivariate dot plots demonstrate expression of CD68^hi^ on brain cells from all groups, and enhanced expression on cells from chronic SD mice. **b** The percentage of phagocytic microglia (CD11b^hi^ CD68^hi^) is significantly elevated in chronic SD mice compared to all other conditions (*p* < 0.001). Results are expressed as mean ± SEM (*n* = 8 per group)

**Fig. 8:**
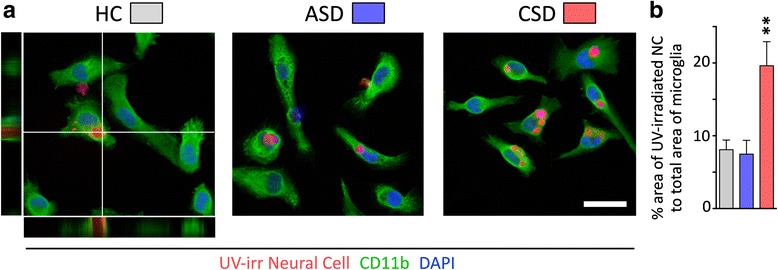
Microglial phagocytic activity is enhanced by chronic social defeat. **a** Representative examples of phagocytosis of neuronal debris are shown. Microglia from homecage (HC) and acute (ASD) or chronic social defeat (CSD) were incubated for 3 h with fluorescently labeled ultraviolet-irradiated neural cells and stained with anti-CD11b. Confirmation of phagocytosis is provided in orthogonal projections of confocal z-stacks shown in the HC picture. Scale bar = 20 μm. **b** Bar graphs compare surface area of ultraviolet-irradiated neural cells (NC) to total surface area (mean ± s.e.m.) of HC, ASD, and CSD microglia (**P < 0.001; one-way ANOVA; representative experiment shown out of three independently performed)

We further asked if stress alters levels of cells presenting CD68, a lysosomal protein associated with phagocytic activity in microglia. This was analyzed by flow cytometry (Fig. 7a). Chronic SD considerably elevated the number of microglia with CD68^hi^ expression compared to HC and acute SD conditions (Fig. 7b; *F*_(2,23)_ = 45.83, *p* < 0.0001). Acute SD had no effect on CD68 expression (Fig. 7b). These flow cytometry findings were buttressed by a functional assay of phagocytic activity of microglia isolated from HC, acute SD, and chronic SD mice. Here, recently isolated microglia were seeded with pre-labeled UV-irradiated neural cells, used as apoptotic targets. The total area of phagocytosed material was compared to the microglia area and used as an index of phagocytic capacity [49]. Chronic SD microglia were observed to have phagocytosed more labeled material compared to all other conditions (*F*_(2,17)_ = 9.95, *p* < 0.005) (Fig. 8).

### Microglial proliferation after acute SD and chronic SD

**Fig. 9:**
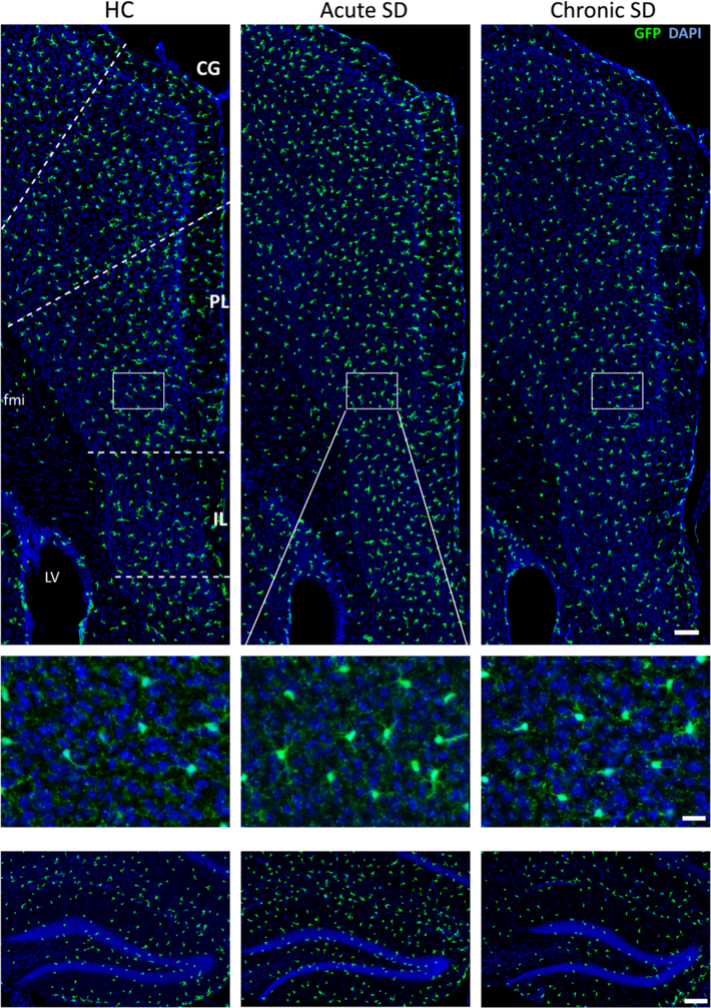
Acute stress substantially increases number of microglia in numerous brain regions of *Cx3cr1* ^*wt/gfp*^ mice. Confocal images showing endogenous GFP expression in the medial prefrontal cortex (*top*) and dorsal hippocampus (*bottom*) of *Cx3cr1* ^*wt/gfp*^ mice exposed to HC, acute SD, or chronic SD. *Insets* show magnified view of prelimbic cortex detailing the endogenous expression of GFP in microglia. Tissue was counterstained with DAPI, shown in *blue. CG* cingulate gyrus, *PL* prelimbic cortex, *IL* infralimbic cortex, *fmi* anterior forceps of the corpus callosum. Scale bars = 100 μm for *top* and *bottom* images and 20 μm for *inset*. A summary of group differences is provided in Table 2; *n* = 8 per group

We hypothesized that SD would increase the number of microglia present within stress-responsive limbic, cortical, and hypothalamic brain regions. Based on previous reports [42, 50, 51], we further hypothesized that changes in mood would inversely correlate with microglia number. We examined the number of microglia from *Cx3cr1*^*wt/gfp*^ mice that were previously phenotyped after exposure to HC, acute SD, or chronic SD (behavioral results discussed in Section 3.1 and data shown in Fig. 1). Surprisingly, whereas the changes in depressive measures occurred only after chronic SD, the stress-induced changes in CX3CR1-GFP+ microglial number occurred only after acute SD (Fig. 9). Acute SD induced a significant increase in the number of microglia in 7 of 12 regions examined, including the infralimbic, prelimbic, and anterior cingulate cortices, piriform cortex, and nucleus accumbens, dorsal dentate gyrus, and basolateral amygdala. Significant declines in microglia cell number were not observed under any condition. Curiously, cell number within the paraventricular nucleus, the master regulator of the stress axis pathway, was not altered by acute SD or chronic SD (Table 2, Fig. 9). We further evaluated whether USM preferences or SI were correlated with microglia counts for each region. However, neither behavior correlated with microglia number in any region (*p* > 0.05).

**Table 2:** Acute stress exposure elevated the number of microglia in numerous brain regions

Area	HC	Acute SD	Chronic SD	ANOVA *F*_(2,23)_
IL	68.75 ± 3.38	87.33 ± 4.2^a^	59.14 ± 1.91	16.71, *p* < 0.001
PL	105.5 ± 3.47	123.66 ± 3.24^a^	99 ± 4.94	9.28, *p* < 0.001
CG	148.72 ± 6.36	193.83 ± 6.83^a^	126.5 ± 4.54	29.11, *p* < 0.001
PIR	75.43 ± 6.19	94.5 ± 2.67^a^	74.75 ± 4.26	5.26, *p* < 0.02
Acb	153.97 ± 2.62	173.96 ± 6.47^b^	128.43 ± 12.44	6.83, *p* < 0.01
dBST	49.29 ± 1.94	45.66 ± 3.71	47.75 ± 2.98	
vBST	60.57 ± 6.29	63.33 ± 2.15	67.87 ± 4.47	
PVN	104.5 ± 8.92	109.28 ± 6.55	117.37 ± 11.34	
Dorsal DG	214.3 ± 10.17	275.4 ± 6.6^a^	219.42 ± 11.54	11.1, *p* < 0.001
BLA	96.43 ± 5.17	148.75 ± 5.28^a^	115 ± 4.85	24.12, *p* < 0.001
DR	88.75 ± 7.82	95.2 ± 3.17	87.4 ± 5.03	
PAG	194.33 ± 9.2	181.6 ± 3.14	167 ± 11.4	

The number of microglia in examined brain regions of CX3CR1GFP/+ mice housed either in homecage (HC) or exposed to social defeat for 3 days (acute SD) or 14 days (chronic SD). Values are expressed as mean ± SEM (*n* = 8 per group). Means with (a) indicate significant difference versus all other groups (*p* < 0.01). Means with (b) indicate significance versus chronic SD samples (*p* < 0.01). *F* values obtained from one-way ANOVAs for microglia number in each brain region

Changes in microglial numbers are likely to come about by local proliferation. To test this directly, a set of experiments probed the origin of microglia in acute SD-exposed animals. PCNA, an endogenous protein largely expressed during S phase of cell cycle, was used to measure cell proliferation. Compared to a 90-min bioavailability window for bromodeoxyuridine (BrdU), PCNA can be detected over an 8-h period, granting more sensitivity without added stress to the animal from multiple injections. Also, BrdU incorporation into microglia could occur through phagocytosis of BrdU-labeled cells and may masquerade as true proliferation.

**Fig. 10:**
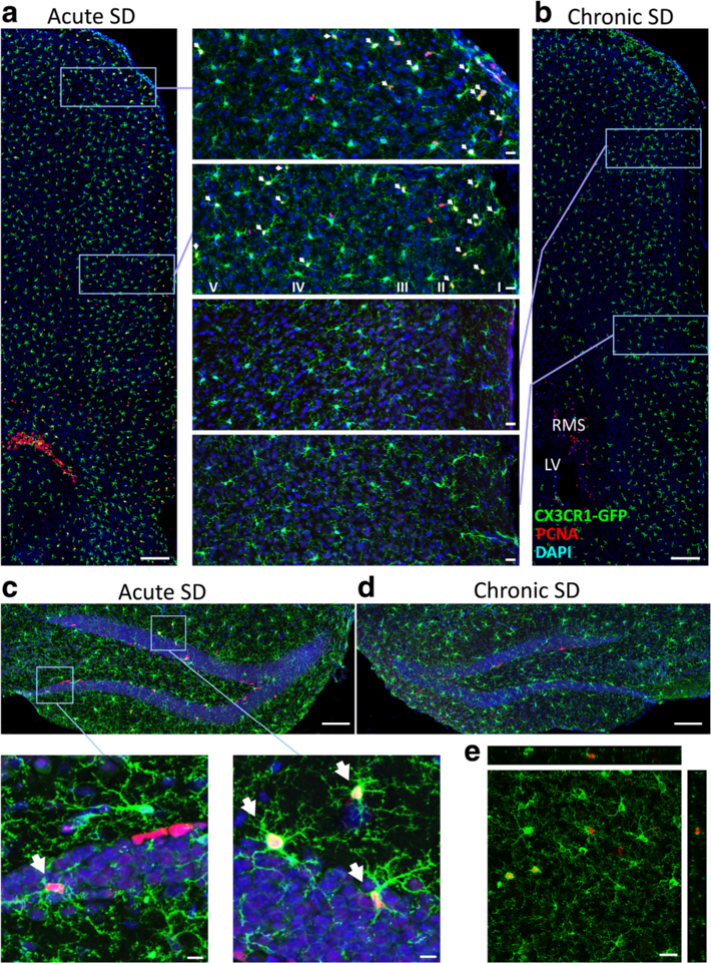
Acute stress exposure increases proliferation of microglia in numerous brain regions of *Cx3cr1* ^*wt/gfp*^ mice. Representative confocal images show that PCNA+ CX3CR1-GFP+ microglia are more prevalent in the medial prefrontal cortex (**a**) and dorsal hippocampus (**c**) of mice exposed to acute stress. **b**, **d** Comparable regions from chronic SD mice. *Inset* panels between (**a**) and (**b**) provide magnified views of PCNA colocalization with GFP microglia and demonstrate the distribution of proliferating cells within cortical layers (I, II, III, IV, and V). Proliferating cells within the rostral migratory stream (RMS) are provided for contrast. *Insets* below **c** show PCNA+ CX3CR1-GFP+ microglia within dentate gyrus and surrounding area. *Arrows* indicate CX3CR1-GFP+ microglia colabeled with PCNA. **e** Example of CX3CR1-GFP+ microglia colabeled with PCNA is shown. Scale bars: **a** and **b** = 200 μm (insets = 20 μm); **c** and **d** = 100 μm (**c** insets = 10 μm); **e** = 20 μm. *LV* lateral ventricle. A summary of group differences is provided in Table 3; *n* = 8 per group

Coincident with the increase in microglial cell numbers, we observed a striking increase in the number of CX3CR1-GFP+ microglia colabeled with PCNA in numerous brain regions of acute SD mice only (Table 3, Fig. 10). Furthermore, structures with elevated microglia cell numbers after acute SD also showed elevated colabeled GFP+ PCNA+ cells. All regions of the medial prefrontal cortex showed elevated GFP+ PCNA+ microglia after acute SD; however, these cells were limited mostly to cortical layers II and III (Fig. 10a, inset). Furthermore, the presence of PCNA+ microglial cells was not completely coupled to structures associated with emotion, as the dorsal and ventral bed nucleus of the stria terminalis (BST) and the PVN—nuclei highly responsive to emotional stimuli—showed no significant changes in microglia cell numbers. Rather, telencephalic but not diencephalic structures showed microglial proliferation.

**Table 3:** Exposure to acute psychological stress substantially elevates microglia proliferation in numerous brain regions

Area	HC	Acute SD	Chronic SD	ANOVA *F*_(2,23)_
IL	0.2 ± .17	23.6 ± 2.27^a^	0	101.7, *p* < 0.001
PL	0.2 ± .17	26.7 ± 2.48^a^	0	109.5, *p* < 0.001
CG	0.4 ± .3	35.1 ± 2.04^a^	0	272.5, *p* < 0.001
PIR	0	21.8 ± 2.44^a^	0.2 ± 0.1	76.88, *p* < 0.001
Acb	0.6 ± 0.36	23.1 ± 2.89^a^	0	57.89, *p* < 0.001
dBST	0	0.7 ± 0.4	0	
vBST	0.25 ± .2	1.2 ± 0.4	0	
PVN	1.0 ± 0.5	7.0 ± 3.0	0.2 ± 0.1	
Dorsal DG	2.7 ± 0.8	19.66 ± 2.1^a^	0.6 ± 0.3	65.13, *p* < 0.001
BLA	0	5.0 ± 1.7^a^	0	8.34, *p* < 0.005
DR	0	7.0 ± 1.1^a^	0	46.67, *p* < 0.001
PAG	1.0 ± 0.4	13.5 ± 2.0^a^	0	40.27, *p* < 0.001

The number of microglia colocalized with a S phase proliferation marker (*PCNA* proliferating cell nuclear antigen) in examined brain regions of CX3CR1GFP/+ mice housed either in homecage (HC) or exposed to social defeat for 2 days (acute SD) or 14 days (chronic SD). Confocal microscopy was used to determine colocalization, and values shown above are expressed as mean ± SEM (*n* = 8 per group). *F* values obtained from one-way ANOVAs for microglia number in each brain region. Means with (a) indicate significant difference versus all other conditions (*p* < 0.01)

## Discussion

### SD stress does not induce monocyte infiltration into the brain

Previous studies have shown that in response to psychological stress, peripheral monocytes infiltrate into the brain where they alter affective behavior and microglia activation (for review, see [52]). Our first question, therefore, was whether a well-characterized form of psychosocial stress—which by design involves minimal physical contact with an aggressor animal and reliably produces profound depressive-like, anxiety-like, and antisocial behaviors [37, 38]—also induces monocyte infiltration. Our first main finding of the study is that acute SD and chronic SD do not elicit monocyte infiltration into the brain. Because it is very difficult to prove a negative result, we approached this question using three distinct methods. First, through flow cytometry, we found the percentage of CD11b^hi^ CD45^hi^ macrophages in the brain was similarly low among all treatment conditions. Second, in an adoptive transfer experiment, GFP-expressing splenocytes were transferred into wildtype mice and tracked in the brain and spleen. Although transferred GFP+ cells successfully colonized wildtype spleen, no cells were detected in the brains of stressed mice. As a positive control, we detected GFP+ cells in the brains of wildtype animals given a strong immune stimulus (i.p. LPS + i.c.v. IL-1β). Third, *Ccr2*^*wt/rfp*^*Cx3cr1*^*wt/gfp*^ transgenic crosses were made to track monocytes in the brain. CCR2, the chemokine receptor that regulates cell mobilization to inflammatory sites, is strongly expressed on inflammatory monocytes [53]. Neither flow cytometry nor immunochemistry revealed the presence of CCR2-RFP+ cells in brain parenchyma after stress. Labeled cells where limited mainly to the meninges and choroid plexus in all animals.

Our finding contrasts with reports of monocyte infiltration and macrophage engraftment following various forms of stress [9–11]. What might be the reason for the discrepancy? First, some studies use a different form of social defeat stress, called repeated social defeat (RSD), in which daily agonistic interactions with the aggressor last for 2 h, after which the aggressor is removed from the cage [10, 45]. In contrast, we use 5 min of agonistic interaction coupled with 24 h of dyadic housing; a combination that contributes powerfully to the overall decline in affect in our studies [34, 36–38] and others [54]. Thus, stressor type and severity may be important. It has been reported that severe stressors, like repeated footshock, elevated cytokine levels in the brain, whereas a form of social defeat did not [55]. Some stressors may invoke a physical or strong physiological component that initiates monocyte trafficking into the brain and the affective sequelae. For example, following liver damage, monocytes enter the brain where they activate microglia and induce depressive-like behavior [56]. Severe pilocarpine-induced seizures that cause neuronal cell death also result in macrophage entry [57]. Cold stress, footshock, and prolonged agonistic interactions possibly associated with wounding might constitute similar kinds of physiological and physical challenges. Indeed, in the RSD model, wounding is a likely explanation for the reported enlarged spleens and glucocorticoid resistance seen in these animals [52]. Engagement of the peripheral immune system might be exacerbated by other environmental factors that vary between laboratories, such as gut microbiota that can shape CNS activity and affective behavior [58]. We here show that psychological stress alone can produce strong effects on affective behavior without triggering monocyte infiltration.

Second, most studies showing stress- or pain-induced monocyte entry into the brain also employ either irradiation or chemotherapeutic agents to create bone-marrow chimeras with labeled hematopoietic cells [9–11, 59]. These treatments can degrade the integrity of the blood-brain barrier [60–63]. In contrast, chimera studies that utilize parabiosis, in which the bloodstream of a GFP-positive partner is connected to a GFP-negative mouse, BM-derived GFP+ peripheral cells are completely absent from the CNS [31, 64]. Thus, irradiation and chemotherapy permit the entry of bone marrow-derived myeloid cells into the tissue. Interestingly, this effect is being exploited for its therapeutic potential. Chemotherapeutics such as busulfan are used to promote transmigration of donor-derived monocytes to the brain [63, 65]. Thus, degradation of the blood-brain barrier may underlie the observed monocyte infiltration and engraftment in stress studies employing irradiation [9, 11, 59] or busulfan treatment [10].

There are several additional noteworthy points. First, we found that CCR2 knockout mice showed chronic SD-induced depressive-like behaviors associated with no monocyte infiltration into the brain. Thus, knocking out the function of CCR2 did not confer behavioral resilience to social defeat stress in this chronic SD model as opposed to the RSD model in which it did [10]. The fact that we see reduced affect in the CCR2 knockout mouse supports our contention that chronic SD manifests the effects of psychological stressors devoid of physiological or pathological content that engages the periphery. Second, we wondered whether monocyte infiltration into the brain might account for the increased microglial cell numbers seen after acute SD. Again, it has been reported that monocytes can enter the brain and assume the morphological characteristics of microglia, but only after irradiation and blood-brain barrier compromise [26, 66]. Thus, the increased cell numbers reflect production of new microglia from local progenitor cells [32], a finding that expands on previous studies [67, 68].

### SD stress does not induce microglial shape changes

We further performed a rigorous quantitative morphological analysis of six features of microglial shape using an automated method [44]. Through this method, we examined 6716 microglia across three stress-responsive brain regions. While we detected no change in microglia morphology after either acute SD or chronic SD, we did observe regionally heterogeneous morphological changes after LPS administration. Profound changes measured in LPS-stimulated animals confirmed that the automated method could detect changes in cell morphology. In each of the three regions examined, LPS elicited noticeable effects on soma size and shape. However, we were surprised to see that hypothalamic microglia were more sensitive to LPS compared to microglia in the cortex and hippocampus. Within the PVN only, we detected significant reductions in cell process length and eccentricity. Relatively few studies have reported microglial responses to inflammatory challenge that vary according to location [69–71]. Changes in microglia morphology in the PVN highlight its heightened immune-alert state compared to other regions, which seems appropriate given the PVN is a major autonomic and neuroendocrine region that regulates sympathetic outflow. Interestingly, the vasculature of the PVN appears to be particularly sensitive to peripheral LPS challenge [72]. SD stress, surprisingly, had little effect on any of the morphological parameters.

These results stand in strong contrast to numerous other studies showing stress-induced changes in microglial morphology, although the direction and actual morphological change reported in these studies have not been consistent. For instance, stress has been reported to both de-ramify and hyper-ramify microglia, to increase and decrease cell size, and to increase and decrease Iba1 levels [33, 45, 50, 73, 74]. Differences may be due to the method used to detect microglia morphology. We choose endogenous GFP in *Cx3cr1*^*wt/gfp*^ mice as our detection and measurement method because there has been no clear demonstration that Iba1 staining reflects true cell morphology or just the intracellular distribution of Iba1 and its particular response to challenge. Indeed, Iba1 is expressed only weakly by resting ramified microglia but strongly by activated microglia [42, 75, 76]. Therefore, Iba1 immunohistochemistry may not capture the finer complexity of resting microglia or the precise changes in cell structure during activation, whereas GFP staining of the entire cell faithfully and passively follows changes in shape. It is important to add that absence of shape changes does not mean the cells are not phenotypically changed, and indeed they are as shown by flow cytometry gating CD68 and the ex vivo phagocytosis assay data.

### Acute SD and chronic SD alter microglial function measured ex vivo

Microglia are normally engaged in clearing debris generated by neural cell death or membrane shedding [23], and we wondered whether this activity is altered under conditions of acute or chronic stress. Elevated numbers of microglia expressing high levels of CD68, a response that characterizes phagocytic macrophages [77], were observed in chronic SD but not acute SD brains. Importantly, elevated CD68 expression detected by flow cytometry also translated functionally—microglia from chronic SD mice showed enhanced phagocytic activity ex vivo. These findings suggest that cellular debris or cell damage or death may be a hallmark of chronic stress effects on the brain. Current experiments are exploring causal links between microglial phagocytic activity, markers of cell stress and damage, and affective behavior in the SD model.

### Acute SD induces region-specific microglial proliferation

Our last main finding is a definitive increase in microglial proliferation following 2 days of acute SD. Levels returned to control levels at 14 days (chronic SD), confirming a similar pattern seen in the hippocampus of mice given a regimen of unpredictable stressors [33]. Microglia expansion occurred in telencephalic areas associated with emotional regulation. No changes were seen in stress-associated subcortical nuclei. It has been suggested that proliferation is a response to elevated glucocorticoids (GCs) in acute stress conditions [68], but the regional specificity of change is a mystery. Microglia can show regional and insult-specific responses [78]. Thus, pockets of proliferation may be where local neural or hormonal signals govern microglial proliferation. Interestingly, voluntary exercise increases microglial proliferation selectively within layers 1–3 of the caudal neocortex [67], a pattern shared with a zone of activity-dependent upregulation of growth factors that support microglial proliferation [79, 80].

If stress-elevated GCs, i.e., corticosterone, are supporting proliferation, then the actions are likely to depend on dose, timing, and context. Very low levels of GCs have been shown to support proliferation whereas higher levels of corticosterone associated with stress exposure [35] are potently anti-proliferative [81]. The development of GC resistance may negate hormone effects in the chronic SD condition. For instance, chronic GC exposure reduces the expression of glucocorticoid-responsive genes such as GILZ (glucocorticoid-induced leucine zipper) and FKBP51 [45]. Many molecules and conditions besides GCs can trigger microglial proliferation and activation (see [17] for review), and the observed results may be due to a combination of stress effectors.

Functionally, there may be strategic advantage to increasing the number of surveying brain immune cells during short-term threat exposure. Enhancement of immune function confers increased protection following wounding or infection that may occur during stress exposure [82, 83]. In contrast, chronic stress is well known to suppress immune function, presumably to protect the host from the detrimental consequences of an overactive inflammatory immune response [1, 84–87]. Thereby, the cumulative impact of chronic stress exposure induces microglial apoptosis [33, 68, 88]. The switch from acute-augmenting to chronic-suppressive stress effects on immune function may occur during the “resistance phase” of the “syndrome of adaptation,” described by Hans Selye [89], in which adaptive processes reinstate homeostasis during stress.

## Conclusions

Chronic psychosocial stress produced through social conflict in a dyadic dominant/subordinate living environment produces an antisocial depressive-like state without infiltration of peripheral monocytes, whose presence in the brain as macrophages is therefore not necessary for either chronic SD-induced altered affect or changes in microglial status. Acute SD was too brief to induce depressive-like behaviors, but it did trigger regionally delineated microglial proliferation, which may result from transient local hormonal changes. In contrast, chronic SD produced a depressive-like state at a time when microglial numbers had reverted to baseline. Neither acute SD nor chronic SD induced changes in microglial morphology, but chronic SD microglia showed elevated levels of CD68 and enhanced phagocytic activity ex vivo, indicating an activation state in the absence of shape changes. Further studies examining microglial diversity and the drivers of this diversity in the context of stress-related mood disorders are warranted. We are currently exploring whether alterations in microglial activation status play a role in the development of stress-induced affective disorders.

## Abbreviations

GFP: Green fluorescent protein
HC: Home-cage
LPS: Lipopolysaccharide
PCNA: Anti-proliferating cell nuclear antigen
PFC: Prefrontal cortex
PVN: Paraventricular nucleus
SD: Social defeat
SI: Social interaction
UBC: Human ubiquitin C
USM: Urine scent marking
